# Contact Angle Measurement of Small Capillary Length Liquid in Super-repelled State

**DOI:** 10.1038/s41598-017-00607-9

**Published:** 2017-04-07

**Authors:** Tingyi “Leo” Liu, Chang-Jin “CJ” Kim

**Affiliations:** 1 Department of Mechanical and Aerospace Engineering, University of California, Los Angeles (UCLA), Los Angeles, California USA; 2 California NanoSystems Institute (CNSI), 570 Westwood Plaza, Los Angeles, CA 90095 USA

## Abstract

The difficulty of measuring very large contact angles (>150 degrees) has become more relevant with the increased popularity of super-repellent surfaces. Measurement is more difficult for dynamic contact angles, for which theoretical profiles do not fit well, and small capillary length liquids, whose sessile droplets sag by gravity. Here, we expand the issue to the limit by investigating dynamic contact angles of liquids with an extremely small capillary length (<1.0 mm), empowered by the superomniphobic surface that can super-repel even fluorinated solvents, which highly wet all materials. Numerically simulating and experimentally testing 13 different liquids on the superomniphobic surface, we discover their dynamic contact angles can be measured with a consistent accuracy despite their vastly different capillary lengths if one keeps the lens magnification inversely proportional to the capillary length. Verifying the droplet equator height is a key parameter, we propose a new Bond number defined by the equator height and optical resolution to represent the measurement accuracy of large contact angles. Despite negligible improvement for most liquids today, the proposed approach teaches how to measure very large contact angles with consistent accuracy when any of the liquids in consideration has a capillary length below 1.0 mm.

## Introduction

Liquid repellent surfaces, especially those providing both very large contact angles (e.g., >150°) and very small contact angle hysteresis (e.g., <10°)^1–8^, have been attracting tremendous attention in recent years. Depending on the liquids they can repel, these surfaces are termed superhydrophobic (i.e., for water), superoleophobic^4^ or superamphiphobic^5^ (i.e., for oils), superomniphobic^6, 7^ (i.e., for all liquids), etc. All dealing with “super-” surfaces have reported a large contact angle in super-repelled state, but many of them have not presented how accurate the measurement was or specified whether the angle was for advancing or receding. Unfortunately, measurement of large contact angles is inherently less accurate (typically undervalued) due to the limited optical resolution near the contact line (i.e., solid-liquid-gas interface) and easily affected by the optical setup and image analysis algorithm employed. This inaccuracy is exacerbated as in some cases the apparent advancing contact angle approaches 180°. Furthermore, the large apparent (i.e., macroscopic) contact angles of interest here have sometimes been mixed up with the temporary (i.e., instantaneous) and local (i.e., microscopic) contact angles viewed right on the microstructures of the super-repellent surfaces, confusing the accuracy of the reported apparent contact angles^9, 10^. The increasing popularity of super-repellent surfaces has made accurate measurement of large contact angles an issue of practical importance^11–13^.

**Figure 1:**
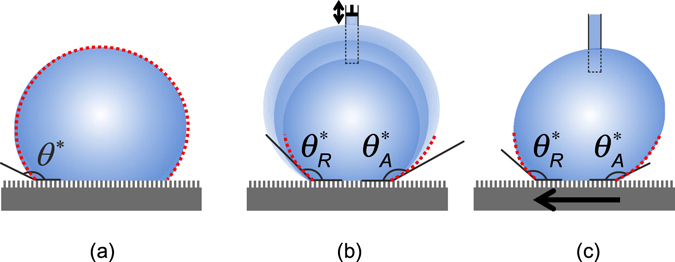
General methods of contact angles measurement, shown on super-repellent surfaces. (**a**) Static contact angle of a symmetric droplet can be measured by fitting the global droplet profile with a theoretical profile calculated from Young-Laplace equation. (**b**) Dynamic contact angles revealed by adding and subtracting the liquid to the droplet through a syringe needle, which tends to compromise the droplet symmetry in practice. (**c**) Dynamic contact angles revealed by sliding the droplet, which destroys the droplet symmetry. For (**b**) and (**c**), which lack the droplet symmetry, dynamic contact angles are usually measured by fitting the local droplet profile near the contact point with a polynomial equation.

Several methods have been studied to measure the large contact angles using a sessile drop. Figure 1a illustrates an experimentally obtained image fitted to the theoretical Young-Laplace equation over the entire profile, allowing one to determine the contact angle more accurately than using the image near the three-phase contact point^11, 12, 14^. Furthermore, assuming axisymmetric interfaces, some advanced profile fitting methods^15, 16^ have eliminated the need to use the apex of the profile, accommodating the common needle-inserted drops. Pendant bubble has also been proposed to increase the accuracy of large contact angle measurement by an order of magnitude, compared with the sessile drop^13^. Because the above methods take the advantage of drop symmetry and can obtain the drop profile from Young-Laplace equation, the accuracy of contact angle is solely determined by the contact line location. However, these global-profile fitting methods are usually not suitable for dynamic contact angles, because the symmetry needed for the theoretical Young-Laplace fitting over the entire profile is easily lost either for a needled droplet subjected to an asymmetric liquid feeding (due to beveled tip, angled insertion, etc.) (Fig. 1b) or for a sliding droplet (Fig. 1c). Consequently, in addition to the contact line location, the detailed drop profile near the contact line is also needed to determine the contact angle, and hence local polynomial fitting near the contact point remains the most common approach to quantify dynamic contact angles with asymmetric sessile drops, providing measurement errors <1°^17^.

An accurate measurement of large contact angles is known to be even more difficult for drops flattened by gravity^10, 12, 18^. Generally, the shape of a droplet is dictated by the dimensionless Bond number *Bo*, which includes a characteristic length of the droplet and the capillary length of the liquid. The capillary length is defined as *l*_cap_ = (*γ* /*ρg*)^1/2^, where *γ* is the liquid surface tension, *ρ* is the liquid density, and *g* is the gravitational acceleration. For a sessile drop, *Bo* can be defined as *Bo* = (*R*_0_/*l*_cap_)^2^, using the radius of curvature at the drop apex (*R*_0_) as the characteristic length^11^. Since for a given volume a liquid with a smaller *l*_cap_ forms a more flattened droplet, one can say it is more difficult to measure the contact angle of a smaller *l*_cap_ liquid in a repelled state^10, 12^. However, to the best of our knowledge, the origin of this difficulty has not been clearly understood largely because few liquids with a small capillary length showed a large contact angle on any solid surface. So far the smallest capillary length liquid studied on a repellent surface was diiodomethane^12^ (*l*_cap_ ≈ 1.3 mm), whose capillary length is ~50% of water’s (*l*_cap_ ≈ 2.7 mm). In this paper, we incorporate the recently introduced superomniphobic surface capable of repelling any existing liquids^7^ to expand the study by covering liquids with an extremely small capillary length, i.e., fluorinated solvents including 3M^TM^ FC-72 (*l*_cap_ ≈ 0.78 mm), whose capillary length is only ~29% of water’s. This true superomniphobic platform allows us, for the first time, to discover a pitfall that has so far been unnoticeable in contact angle measurements, especially on super-repellent surfaces.

In this paper, we first theoretically investigate the origin of the difficulty in measuring large contact angles of gravity-flattened drops using numerical simulations. The result exposes the common pitfall and explains the difficulty in accurately measuring large contact angles. Based on the simulation, we conclude that a lens magnification corresponding to the ratio of the capillary lengths should be used in order to obtain values of a consistent accuracy when measuring large contact angles of liquids with significantly different capillary lengths. To confirm the effectiveness of the proposed rule, we will examine the dynamic contact angles of 13 liquids with widely different capillary lengths on a superomniphobic SiO_2_ surface, on which all the liquids have the same apparent contact angles, reconciling the inaccurate contact angles obtained before adopting the proposed measurement rule. By generalizing the findings, along the way we define a new Bond number based on the equator height of beading droplets and the resolution of the optical system to quantify the measurement accuracy of large contact angles.

## Theory

**Figure 2:**
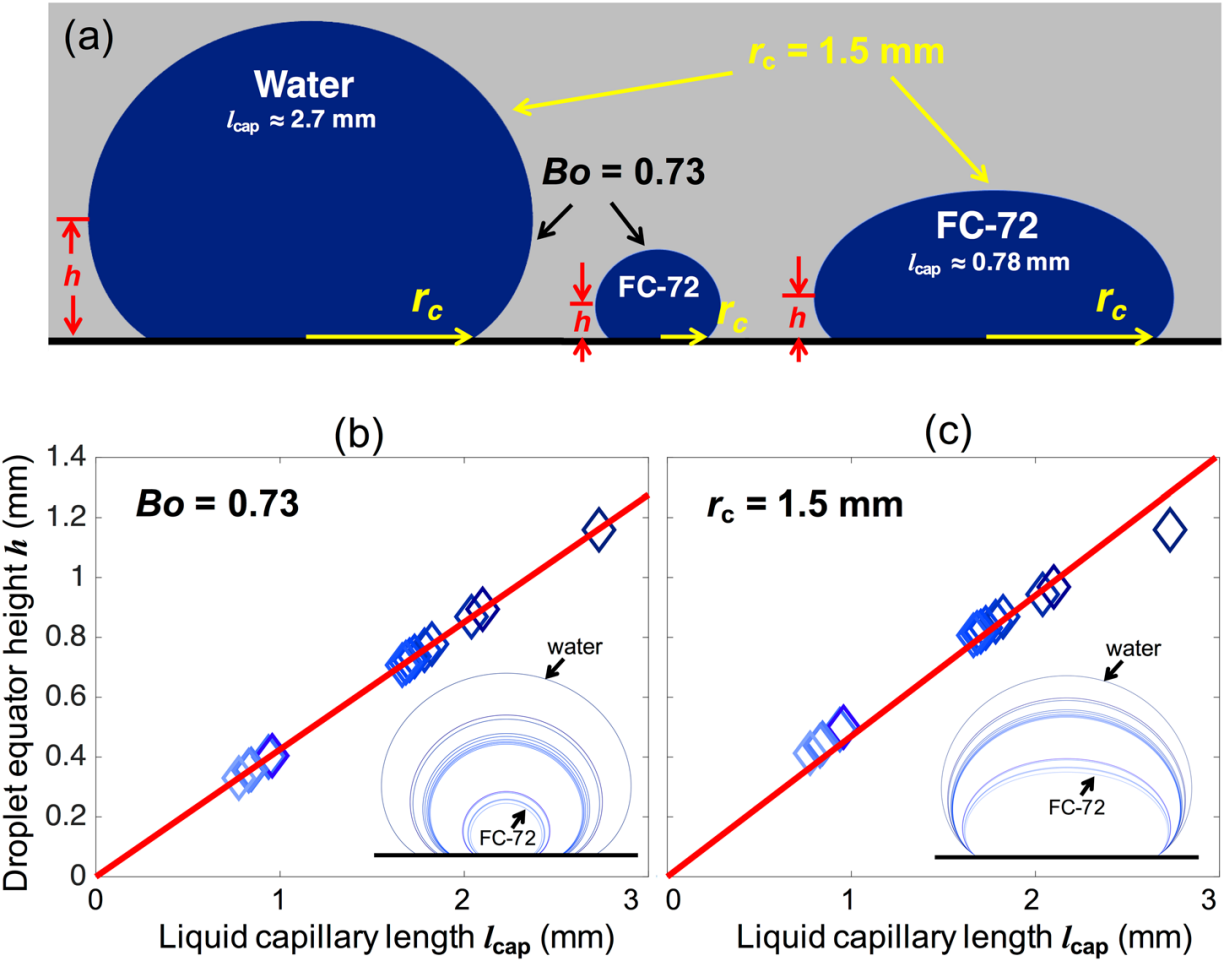
Sessile drops of two liquids with very different capillary lengths *l*_cap_ and same large contact angle. (**a**) Droplet profiles of water and FC-72 drawn from numerical integration with apparent contact angle *θ* ^*^ = 150°. Comparing the water droplet and the small FC-72 droplet in the middle, they share the same Bond number (*Bo* = 0.73) and thus the same profile shapes, but the FC-72 droplet is smaller due to its smaller *l*_cap_. Comparing the water droplet and the large FC-72 droplet on the right, they share the same contact radius (*r*_c_ = 1.5 mm), but the FC-72 droplet is flattened more due to its smaller *l*_cap_. Now, comparing the two FC-72 droplets, notice their equator heights *h* are similar despite a large difference in their volumes. This departure between *h*, which determines the image resolution, and the droplet size, which is commonly used to decide the resolution, leads to the common inaccuracy, which we propose to correct. (**b** and **c**) Relation between *h* and *l*_cap_ from the theoretical profiles of 13 different liquids selected for experiments in this paper. The red lines show the linear fitting. The insets show the droplet profiles under their respective boundary conditions. In (**b**), water and FC-72 are compared with *Bo* = 0.73, i.e., the left and middle droplets in (**a**), over 0.42 mm < *r*_c_ < 1.5 mm. In (**c**), water and FC-72 are compared with *r*_c_ = 1.5 mm, i.e., the left and right droplets in (**a**), over 0.73 ≤ *Bo* ≤ 17.

For contact angle measurements, a common practice is to capture a droplet image at a magnification that will fill the picture frame fully with the image to maximize optical resolution, i.e., minimize pixel size. Figure 2 is drawn to help explain a common problem in measuring the contact angle of a very small capillary length liquid in a repelled state. Figure 2a depicts theoretical profiles of sessile drops of water and FC-72 numerically integrated from Young-Laplace equation^19^ assuming a constant apparent contact angle *θ* ^*^ = 150°. Here we introduce a term called droplet equator height (*h*), which is defined as the height of the largest horizontal section of the drop above the tested surface. The equator height here is different from the equatorial height used in Bashforth and Adams^20^ and Padday^21^, as explained in Figure S1 in the Supplementary Information. Figure 2b and c show the droplet equator heights calculated for 13 different liquids by fixing their droplet shape and landing area, respectively.

When measuring the contact angles of droplets with a similar *Bo* but different sizes, e.g., water droplet on the left and the small FC-72 droplet in the middle in Fig. 2a, one would fill the picture frame with the droplet image, ending up using a lower lens magnification for water than for FC-72. While viable for smooth surfaces^22^ as far as the lens has a high enough magnification, this practice has a limitation for super-repellent surfaces, because contact angle measurement becomes unreliable if the droplet is not much larger than their roughness scale, e.g., pitch or periodicity of the surface microstructures^23^. This limitation is relevant because often water droplets of just a few millimeters in diameter are placed on a superhydrophobic (SHPo) surface whose structural pitch is not much smaller than 1 mm. Since FC-72 droplets with a similar shape as the water droplet (e.g., the small FC-72 droplet in the middle) may land on only several structures, one will increase their volume until their contact radius (*r*_c_) becomes similar to the water drop’s as shown in the large FC-72 droplet on the right. Since the large FC-72 droplet has roughly the same maximum diameter as the water droplet, one will use the same lens magnification as the water droplet when capturing images for contact angle measurements. This is how the inaccuracy starts. Note the large FC-72 droplet on the right has a similar equator height (*h*) as the much smaller FC-72 droplet in the middle. As result, the above casual (albeit natural) lens selection practice was essentially equivalent to capturing the small FC-72 droplet with the same lens magnification used for the large water droplet, ending up analyzing one droplet profile (i.e., same *Bo*) with two different resolutions (i.e., low resolution for FC-72). This exercise explains why the contact angles on gravity-flattened droplets are commonly measured less accurately.

From Fig. 2a, we have noted the proper parameter representing the measurement accuracy of large contact angles is the equator height (*h*) since it indicates the lens magnification needed reliably. To estimate the magnification for different liquids, we plotted *h* versus *l*_cap_ for 13 different liquids with the same droplet shape (*Bo* = 0.73) in Fig. 2b and the same contact radius (*r*_c_ = 1.5 mm) in Fig. 2c, showing a good linear relationship for both cases. See Figure S2 in the Supplementary Information for more details. Based on Fig. 2c (and Figure S3 in the Supplementary Information for a similar scaling in the lateral direction), we conclude that one should use a lens magnification inversely proportional to the capillary length to obtain large contact angles (i.e., in super-repelled state) with a consistent accuracy for different liquids over a wide range of capillary lengths.

Now, let us expand the above lens selection rule to see if we can generalize it and quantify the measurement accuracy of large contact angles. The discussion with lens magnification can be further understood in terms of the physical length per pixel. Since we want to fill the picture frame (containing the number of pixels (*N*)) with the image up to *h*, for a given optical system (hence a given maximum *N*) the physical length per pixel (*h*/*N*) is proportional to *h*, meaning larger magnification (hence smaller *h*/*N*) for droplets with smaller *h*. Using this *h*/*N* as the characteristic length, here we define a new Bond number *Bo*_*h*/*N*_ = (*h*/*N*/*l*_cap_)^2^, which quantitatively defines the measurement accuracy of large contact angles. Note the characteristic length *h*/*N* in *Bo*_*h*/*N*_ represents an experimental parameter (*N*) as well as a physical parameter of the droplet (*h*), unlike the usual Bond numbers, whose characteristic length represent only a physical parameter of the droplet (e.g., *R*_0_). When measuring contact angles of liquids with widely different capillary lengths on a repellent surface, *Bo*_*h*/*N*_ should be kept constant to prevent insufficient image resolution prone to small capillary length liquids. While the highest possible lens magnification would provide the most accurate contact angles for a given liquid, the lens selection with a constant *Bo*_*h/N*_ would produce contact angles of different liquids with a consistent accuracy. This consistency is important for a fair comparison when liquids with significantly different capillary lengths are to be discussed in terms of their large contact angles.

## Results and Discussion

**Figure 3:**
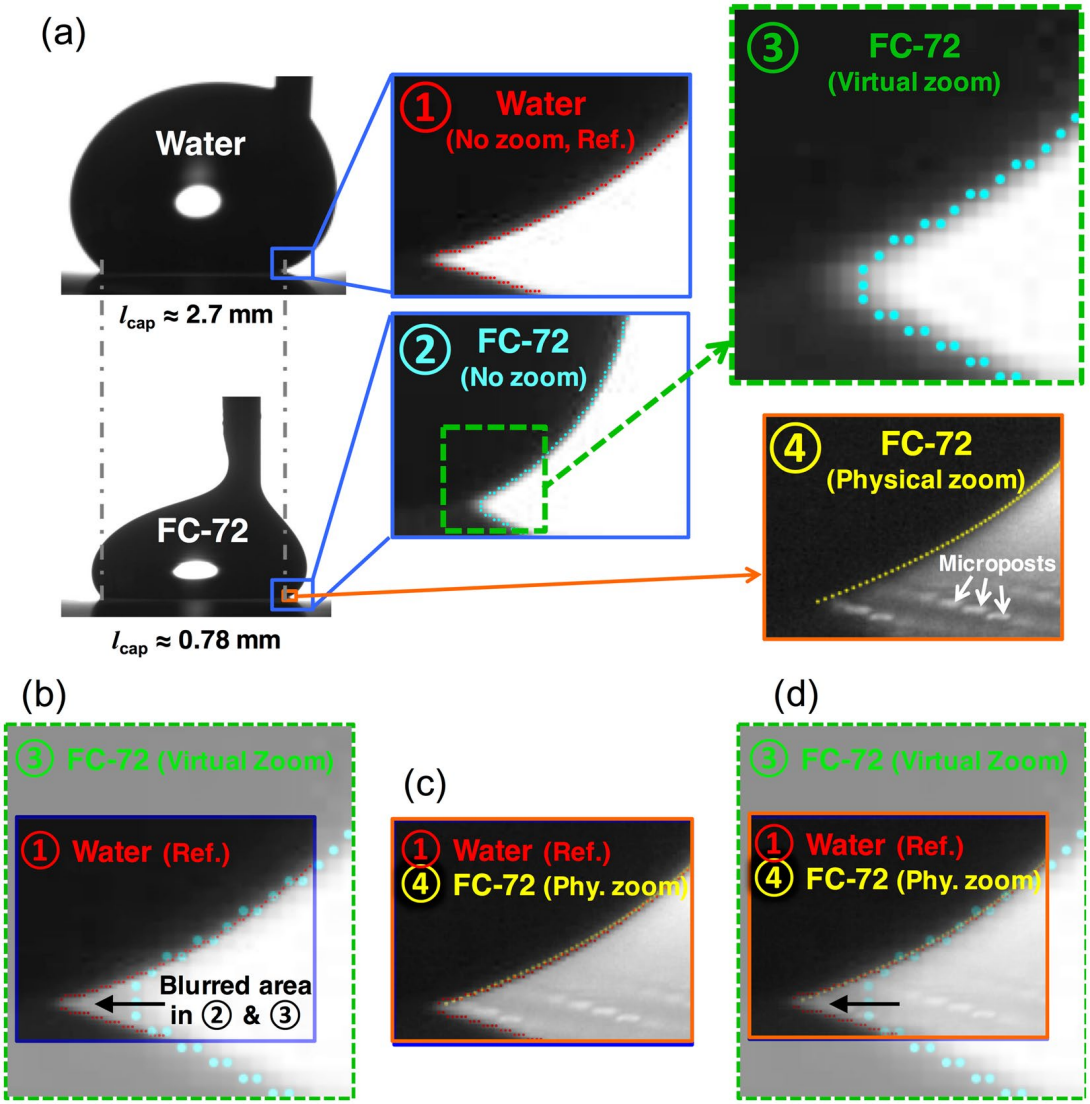
Experimental profiles of water and FC-72 at different magnifications and their comparisons. (**a**) Whole droplet profiles of water and FC-72 and different zooms at their advancing menisci. ① and ② show the advancing menisci of water and FC-72 close to the contact point at no zoom. ③ and ④ demonstrate the advancing meniscus of FC-72 under virtual and physical zoom set to be inversely proportional to the ratio of its capillary length to water. (**b**) Overlap of ① and ③ shows the matching of water and FC-72 (virtual zoom) advancing menisci except the blurred region in FC-72 due to limited resolution and lighting. (**c**) Overlap of ① and ④ shows the matching of water and FC-72 (physical zoom) advancing menisci with the blurred region resolved by higher magnification. (**d**) Overlap of ①, ③ and ④ summarizes the conclusions from (**b**) and (**c**).

**Figure 4:**
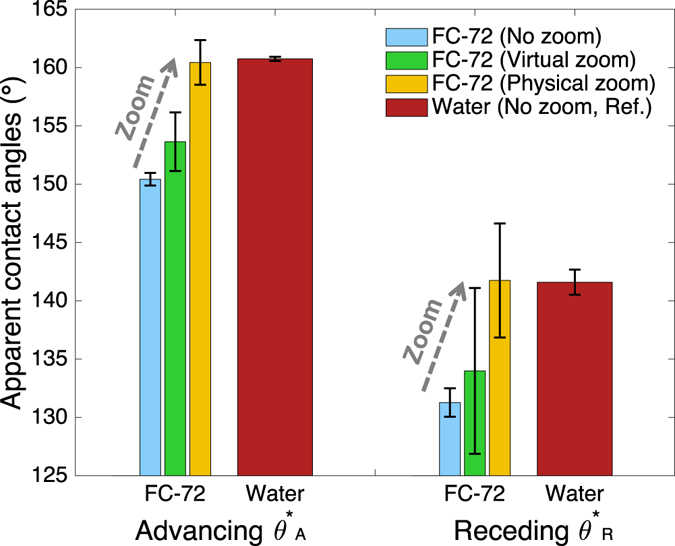
Comparisons of measured contact angles of FC-72 at different zooms with water as a reference. Without zoom, there is ~10° contact angle deviation of FC-72 due to blur that causes an inaccurate meniscus detection. With virtual zoom, the image was digitally scaled but provides fewer points for calculation, leading to more scattered data. With physical zoom, the meniscus was better resolved and showed accurate contact angle while the data scattering was caused by local contact angle variation from a relatively large wavelength of the surface roughness.

Using the advancing menisci of water and FC-72 as examples, Fig. 3a shows the captured images at different zoom levels. In Fig. 3a, ① and ② present the original digital image (i.e., no zoom) near the contact line of water and FC-72, respectively, while ③ and ④ depict the virtual (from ②) and physical zoomed images of FC-72. By overlapping the virtually zoomed image of FC-72 with water, i.e., overlapping ③ on ①, in Fig. 3b we found that the limited resolution near the contact point of FC-72 (③) caused the edge detection of the meniscus to truncate the wedge prematurely. However, when overlapping the physically zoomed image of FC-72 with water, i.e., overlapping ④ on ①, in Fig. 3c we found their menisci completely overlapped. This exercise confirms that the blur in images of FC-72 at no zoom (②) are simply bad lighting through a small gap under the large contact angles formed on super-repellent surfaces. In Fig. 3d, ①, ③ and ④ are overlapped to further illustrate the reconciliation between the contact angle of water and that of FC-72 at virtual and physical zoom.

Figure 4 shows the calculated dynamic contact angles of water and FC-72 at different zooms and confirms that the contact angles of FC-72 have been better resolved by zooming. With virtual zoom, the menisci for FC-72 have fewer points available for the contact-angle calculation, resulting in more scattered data. With physical zoom, the menisci of FC-72 are captured at a sufficiently high resolution, resulting in values close to water contact angles, which are deemed highly accurate. Note that when we measured water contact angles at the same high magnification used for FC-72 under physical zoom (i.e., ~8.76x), the advancing and receding apparent contact angles were measured to be 160.7 ± 0.8° and 139.1 ± 1.7° (mean ± standard deviation, *N* > 500), respectively, which are almost the same as the ones measured at no zoom (160.7 ± 0.2° and 141.5 ± 1.1°). This invariance supports the effectiveness of the magnifications listed in Table 1 and confirms it does not help much to increase the lens magnification further. Instead, too high a magnification may hurt the measurement, because on structured surfaces the intrinsic (i.e., microscopic) contact angles on the local surfaces of individual microstructures start to affect the measurement meant for the apparent (i.e., macroscopic) contact angles^24, 25^. Indeed, the increased scatter of the data in our measurement at very high magnifications indicates that the measurement was performed too close to the structured surface (i.e., a length scale approached the pitch of the micro-posts^24^), as evident from the individual micro-posts appearing in Fig. 3a ④). Elimination of such scatter for physically zoomed data would require a superomniphobic SiO_2_ surface with smaller pitches (e.g., <10 μm), which is not practical, or use of a lower magnification lens, which lowers the measurement accuracy. Note that, while an incorrect determination of the contact point will in general lower the accuracy of the measured contact angles^11, 13^, for the current study the inaccuracy caused by an incorrect contact point determination is negligible (e.g., <1° even for 5 pixels offsets; see Figure S4 in the Supplementary Information) compared with the inaccuracy by a wrong lens magnification (~10°).

**Table 1:** Properties of the test liquids with their capillary length and magnification used for an accurate contact angles measurement.

Name	Chemical Formula	Surface Tension (mN/m)	Density (kg/m^3^)	Capillary length (mm)	Lens Magnification
Water	H_2_O	72.8	997	2.73	2.50 (Ref.)
[EMIM][BF_4_]	C_6_H_11_BF_4_N_2_	52.8	1294	2.04	3.34
Diiodomethane	CH_2_I_2_	50.8	3325	1.25	N/A
Ethylene glycol	C_2_H_6_O_2_	48.2	1113.5	2.10	3.25
Formic acid	CH_2_O_2_	38	1223	1.78	3.83
Toluene	C_7_H_8_	28.3	866.8	1.82	3.74
Acetone	C_3_H_6_O	23.1	784.5	1.73	3.94
Methanol	CH_4_O	22.5	791.4	1.70	4.00
2-Propanol	C_3_H_8_O	21.2	780.9	1.66	4.01
Hexane	C_6_H_14_	18.4	660.6	1.68	4.05
Novec 7100	C_4_F_9_OCH_3_	13.6	1520	0.96	7.14
FC-84	C_7_F_16_	12	1730	0.84	8.11
Novec 649	C_6_F_9_O	10.8	1600	0.83	8.22
FC-72	C_6_F_14_	10	1680	0.78	8.76

Diiodomethane is also included as a comparison^12^.

**Figure 5:**
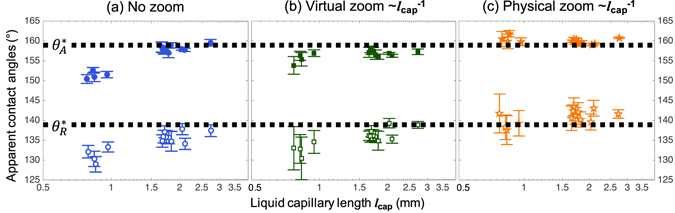
Contact angles of 13 different liquids measured on a superomniphobic SiO_2_ surface at different zooms with the accurate values shown as the horizontal dotted lines. (**a**) With no zoom, liquids with very small capillary lengths (fluorinated solvents) show deviations with their contact angles consistently underestimated. (**b**) Virtual zoom helps mitigating the deviation without readjusting the experiment setups. (**c**) With physical zoom and the proposed rule, the contact angles are obtained with a consistent accuracy regardless of the liquids. The dashed lines indicate the apparent contact angles measured with physical zoom and are drawn as visual guides for comparison with (**a**) and (**b**).

Contact angles have been measured for the 13 test liquids under different zooming conditions and summarized in Fig. 5. As seen in Fig. 5a, the contact angles are significantly undervalued for liquids with capillary length below 1 mm, which are the fluorinated solvents tested for the first time in this report. Other liquids, including water and organic solvents, do not show much error even without corrected magnification, indicating that contact angles reported on usual super-repellent surfaces were generally acceptable. This is because the density and surface tension counter-balance each other for common liquids, including water, organic solvents, and liquid metals^26^, giving them similar capillary lengths (*l*_cap_ > 1.5 mm). In other words, the corrected magnification above is not needed for large contact angles (i.e., >150°) unless the samples include extremely small capillary length liquids (i.e., *l*_cap_ < 1.0 mm). Figure 5b and c show the effectiveness of proper zooming in improving the contact angle measurement for small capillary length liquids. The virtual (i.e., digital) zooming is less accurate but more convenient, while the physical zooming requiring readjustment of the existing setup provides more accurate contact angles. The polynomial fitting was found reliable for all physical zooms used in our system (i.e., coefficient of determination *R* ^2^ > 0.997 for all measurements; see Figure S5 in the Supplementary Information).

## Methods

To verify our theory and demonstrate the effectiveness of our proposal, we utilize a recently developed superomniphobic SiO_2_ surface^7^, which has two unparalleled properties ideal for the current study: (1) The surface is capable of super-repelling all liquids, including those with extremely small capillary lengths; (2) The apparent contact angle on the surface is theoretically identical to all liquids, allowing us to evaluate the accuracy of different measurement methods. The second property was possible because, unlike any other super-repellent surface, the superomniphobic SiO_2_ surface was designed to repel liquids purely by the shape of the surface structures. This insensitivity to both the substrate material and liquid material often invites one to call it a “mechanical surface”. Since all the liquids have indistinguishably small intrinsic contact angles (i.e., <10°) on SiO_2_ and share the same solid fraction (~5%) of the surface structures, there is no difference in the apparent contact angles between different liquids on the superomniphobic SiO_2_ surface as predicted by the Cassie-Baxter model^27^. In contrast, the apparent contact angle on a typical liquid-repellent surface, invariably made of a hydrophobic material, is inevitably affected by the intrinsic contact angle of the liquid and the resulting solid fraction. For example, a liquid with smaller surface tension would exhibit a smaller apparent contact angle, because its intrinsic contact angle is smaller and the solid fraction larger as the small-contact-angle liquid penetrates the roughness more.

All the measurements have been made on the superomniphobic SiO_2_ surface composed of a square array of circular posts (20 μm diameter and 100 μm pitch) capped with doubly re-entrant overhangs^7^. To avoid the spatial effect^24^ of a droplet with size comparable to the wavelength (i.e., pitch) of the roughness^23^ and maintain a consistent heterogeneity condition, we created droplets of different liquids with a similar base radius (*r*_c_ ~1.5–2.0 mm). Advancing and receding contact angles were measured on a custom-built goniometer on a vibration-isolation plate (Vistek VIP Series 320), which consists of an X-Y axis positioning stage amid observation instruments (GO^®^ Edmund VZM™ 1000i Zoom Imaging Lens at 2.5x-10x magnification with Point Grey FL3-U3–13Y3M-C CMOS camera) and a light source. Droplets were created and held by a syringe (BD PrecisionGlide Needle, 22 G × 1.5 inch) on the superomniphobic SiO_2_ surface (Fig. 3a), which was placed on a linear-motorized stage (Zaber T-LA28A-S), so that an advancing and a receding meniscus are formed at the two ends of the droplet by sliding the stage (~200 µm/s). Using water as a reference (at 2.5x), the lens magnification for each liquid was set to the ratio of its capillary length to the water’s, as summarized in Table 1. Note that the 2.5x magnification in our setup provides enough resolution to measure *θ* ^*^ for water (i.e., *Δθ* ^*^ < 1° at ±1 pixel detection uncertainty). All contact angles were measured from the same direction (11.25°, see Figure S6 in Supplementary Information for more details) relative to the grid array of the posts so that the measurements are not affected by viewing directions^24^. For each image, the profile of the droplet was detected by Canny edge detection algorithm^17^ and the profile near the contact point was fitted to quartic polynomial curves^17^. The advancing and receding contact angles were then calculated from the slopes of the curves at the contact point.To compare, we have tested both a virtual zoom and a physical zoom for contact angles measurement. For virtual zoom, we first captured the images at the same magnification for all liquids (i.e., at 2.5x) and then virtually (digitally) scaled the recorded images according to their capillary lengths. For physical zoom, we adjusted the lens magnification accordingly to their capillary lengths (i.e., Table 1) before capturing the images of advancing and receding ends.

## Summary and Conclusions

Exposing a hidden pitfall in measuring very large contact angles (i.e., in super-repelled state) by expanding the tested liquids to those with extremely small capillary length (<1.0 mm), we have developed a practical remedy that ensures a consistent measurement accuracy over different capillary lengths. When measuring the contact angles of small capillary length liquids, we have found one tends to unintentionally capture images with a lower resolution than possible. Demonstrating that the droplet equator height is the key parameter to accurately measure large contact angles using the images near the contact point, we have proposed a Bond number based on the droplet equator height and optical resolution. This new Bond number represents the measurement accuracy of large contact angle for a wide range of liquids. To apply the proposed rule, we used lens magnification inversely proportional to the capillary length of the test liquid and confirmed its effectiveness through experimental verification. The proposed method allows one to avoid underestimating large contact angles (>150°) of very small capillary length liquids (<1.0 mm) compared with those of common liquids (>1.5 mm). While acknowledging the measurement improvement by the proposed method would be negligible for most liquids, it sheds a light to the difficulty associated with optical measurements of very large contact angles accurately. We suggest utilizing this relatively simple practice for a fair comparison of different liquids in terms of large contact angles, if some of the liquids have a very low energy, which tend to have a very small capillary length.

## Electronic supplementrary material

Supplementary Information

## References

[1] Onda T, Shibuichi S, Satoh N, Tsujii K (1996). Super-Water-Repellent Fractal Surfaces. Langmuir.

[2] Kim, J. & Kim, C.-J. Nanostructured surfaces for dramatic reduction of flow resistance in droplet-based microfluidics. In *Proc. IEEE Conf. MEMS* 479–482, doi:10.1109/MEMSYS.2002.984306 (2002).

[3] Gao L, McCarthy TJ (2007). A Commercially Available Perfectly Hydrophobic Material (θ_A_/θ_R_=180°/180°). Langmuir.

[4] Tuteja A (2007). Designing superoleophobic surfaces. Science.

[5] Deng X, Mammen L, Butt H-J, Vollmer D (2012). Candle Soot as a Template for a Transparent Robust Superamphiphobic Coating. Science.

[6] Pan S, Kota AK, Mabry JM, Tuteja A (2013). Superomniphobic Surfaces for Effective Chemical Shielding. J. Am. Chem. Soc..

[7] Liu T, Kim C-J (2014). Turning a surface superrepellent even to completely wetting liquids. Science.

[8] Lu Y (2015). Robust self-cleaning surfaces that function when exposed to either air or oil. Science.

[9] Schellenberger F, Encinas N, Vollmer D, Butt H-J (2016). How Water Advances on Superhydrophobic Surfaces. Phys. Rev. Lett..

[10] Extrand CW, Moon SI (2010). Contact Angles of Liquid Drops on Super Hydrophobic Surfaces: Understanding the Role of Flattening of Drops by Gravity. Langmuir.

[11] Hung Y-L, Chang Y-Y, Wang M-J, Lin S-Y (2010). A simple method for measuring the superhydrophobic contact angle with high accuracy. Rev. Sci. Instrum..

[12] Srinivasan S, McKinley GH, Cohen RE (2011). Assessing the Accuracy of Contact Angle Measurements for Sessile Drops on Liquid-Repellent Surfaces. Langmuir.

[13] Ling WYL, Ng TW, Neild A (2011). Pendant Bubble Method for an Accurate Characterization of Superhydrophobic Surfaces. Langmuir.

[14] Yuan, Y. & Lee, T. R. In *Surface Science Techniques* (eds Bracco, G. & Holst, B.) 3–34, (Springer Berlin Heidelberg, 2013), doi:10.1007/978-3-642-34243-1_1.

[15] Cabezas MG, Bateni A, Montanero JM, Neumann AW (2006). Determination of Surface Tension and Contact Angle from the Shapes of Axisymmetric Fluid Interfaces without Use of Apex Coordinates. Langmuir.

[16] Kalantarian A, David R, Neumann AW (2009). Methodology for High Accuracy Contact Angle Measurement. Langmuir.

[17] Chini SF, Amirfazli A (2011). A method for measuring contact angle of asymmetric and symmetric drops. Colloids Surf. Physicochem. Eng. Asp..

[18] Dorrer C, Rühe J (2008). Some thoughts on superhydrophobic wetting. Soft Matter.

[19] Adamson, A. W. & Gast, A. P. *Physical Chemistry of Surfaces*. (Wiley-Interscience, 1997).

[20] Bashforth, F. & Adams, J. C. An attempt to test the theories of capillary action by comparing the theoretical and measured forms of drops of fluid. *With an explanation of the method of integration employed in constucting the tables which give the theoretical forms of such drops*. (Cambridge [Eng.] University Press, 1883).

[21] J. F. Padday. In *Surface and Colloid Science* (ed. E. Matijević) **1**, 39–248 (Wiley, 1969).

[22] Extrand CW, Moon SI (2010). When Sessile Drops Are No Longer Small: Transitions from Spherical to Fully Flattened. Langmuir.

[23] Marmur A, Bittoun E (2009). When Wenzel and Cassie Are Right: Reconciling Local and Global Considerations. Langmuir.

[24] Liu T, Chen Z, Kim C-J (2015). A dynamic Cassie–Baxter model. Soft Matter.

[25] Zhang J, Kwok DY (2006). Contact Line and Contact Angle Dynamics in Superhydrophobic Channels. Langmuir.

[26] Liu T, Sen P, Kim C-J (2012). Characterization of nontoxic liquid-metal alloy Galinstan for applications in microdevices. J. Microelectromechanical Syst..

[27] Cassie A, Baxter S (1944). Wettability of porous surfaces. Trans. Faraday Soc..

