# Supplementary material for: Contact Angle Measurement of Small Capillary Length Liquid in Super-repelled State
Source: Sci Rep. 2017 Apr 7;7:740. doi: 10.1038/s41598-017-00607-9 (PMC5428877; doi:10.1038/s41598-017-00607-9)
Supplement: Supplementary file 1 — Supplementary Information [file 41598_2017_607_MOESM1_ESM.pdf]

Supplementary Information

Contact Angle Measurement of
Small Capillary Length Liquid in Super-repelled State

Tingyi "Leo" Liu[*†] *& Chang-Jin "CJ" Kim*

Department of Mechanical and Aerospace Engineering
University of California, Los Angeles (UCLA), California 90095, USA

This PDF file includes:

Supplementary Text: Difference between equator height (h) and equatorial height (H)

Figure S1. Definition of equatorial height H and its relations with capillary length l_{cap}

Figure S2. Relation between droplet equator height h and contact radius r_c, numerically simulated for a liquid with capillary length l_{cap} = 0.78 mm (e.g., FC-72) and apparent contact angle θ^* = 150° (i.e., super-repelled). All the symbols correspond to the droplet profiles drawn in the inset, one to one.

Figure S3. Difference between droplet equator radius and contact radius (r_{max} - r_{c}) vs. capillary length l_{cap} when the contact radius and apparent contact angle are fixed at r_{c} = 1.5 mm and θ^* = 150°.

Figure S4. Effect of different contact point selections on the measured apparent contact angles. Images of FC-72 droplets captured at high magnification are analyzed.

Figure S5. Goodness of polynomial fitting vs. physical length per pixel (h/N) of all physical zoomed apparent contact angle data.

Figure S6. Determination of the best viewing angle to measure the apparent contact angle on a superhydrophobic surface consisting of a square-array of micro-posts.

Supplementary Text

Difference between equator height (h) and equatorial height (H)

The equator height in this paper is different from the equatorial height of *Bashforth and Adams*[20] and *Padday*[21] defined as the height of the vertex (apex) of a large drop above the largest horizontal section of the drop, i.e., H shown in Figure S1(a). Unlike the equator height h, the equatorial height H does not have an approximate linear relationship with liquid capillary length for different liquid droplets with a same contact radius, as revealed in Figure S1(c). Instead, as shown in Figure S1(b), H is proportional to the capillary length for different liquids with a same Bond number, which is the unwritten assumption of the methods described by *Padday*[21].

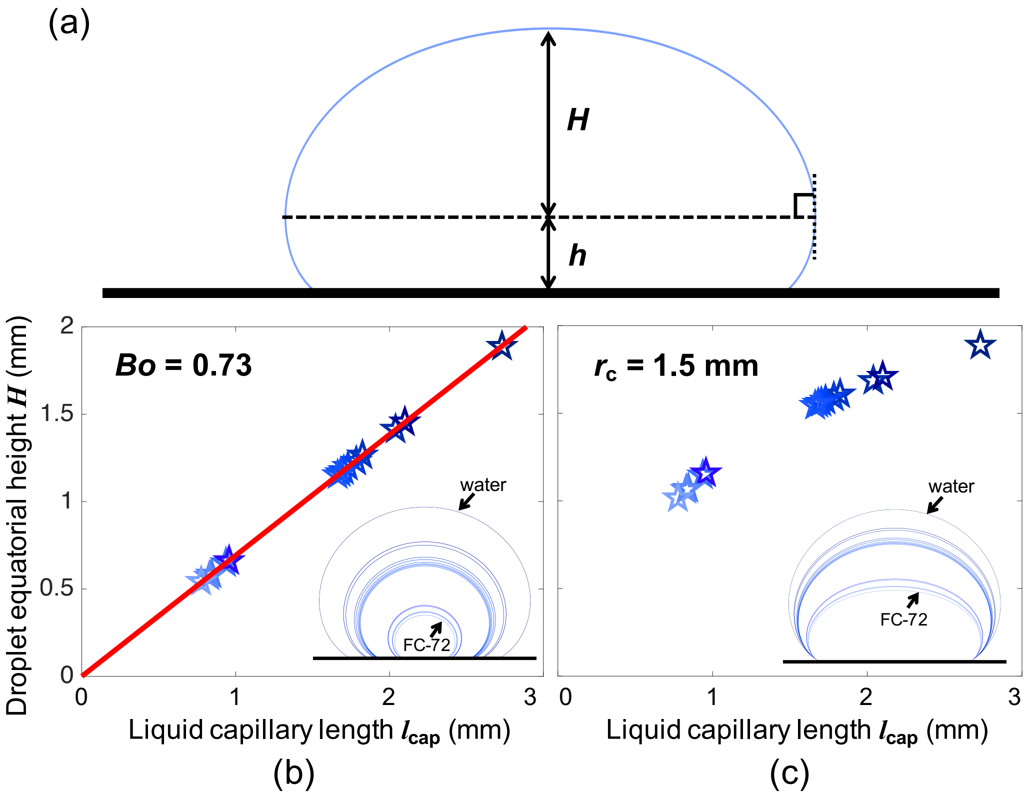

Figure S1. Definition of equatorial height H and its relations with capillary length l_{cap}. (a) Definitions of the equator height h defined in this paper and the equatorial height H used in *Bashforth and Adams*[20] and *Padday*[21]. (b) Relations of H to capillary length (l_{cap}) for liquids with a fixed Bond number (Bo = 0.73). Sharing a same Bo, all liquids have all dimensions proportional to l_{cap}, including H showing here and h shown in Figure 2b. (c) Relations of H to capillary length (l_{cap}) for liquids with a fixed contact radius/area. Unlike h that was shown to have a linear relation to l_{cap} in Figure 2c in the main text, H does not show a linear relation to l_{cap}.

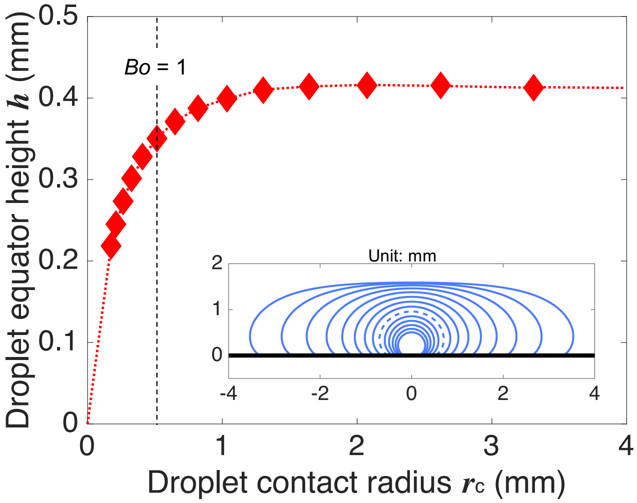

Figure S2. Relation between droplet equator height h and contact radius r_c, numerically simulated for a liquid with capillary length l_{cap} = 0.78 mm (e.g., FC-72) and apparent contact angle θ^* = 150° (i.e., super-repelled). All the symbols correspond to the droplet profiles drawn in the inset, one to one. Both h and r_c increase with the droplet volume, but h slows its increasing rate as the gravity starts to play a role. Once a droplet is heavily flatted by gravity (i.e., $Bo > 1$; e.g., more than ~ 1.1 μL of FC-72), its equator height would remain similar to that ($h \sim 0.4$ mm) of a spherical droplet (i.e., $Bo \approx 1$, whose profile is drawn with a dashed line). The profiles show h peaking at ~0.4 mm but not changing much for r_c greater than ~1.5 mm. Note that Bo is defined as $Bo = (R_0 / l_{\mathrm{cap}})^2$, where R_0 is the radius of curvature at the apex.

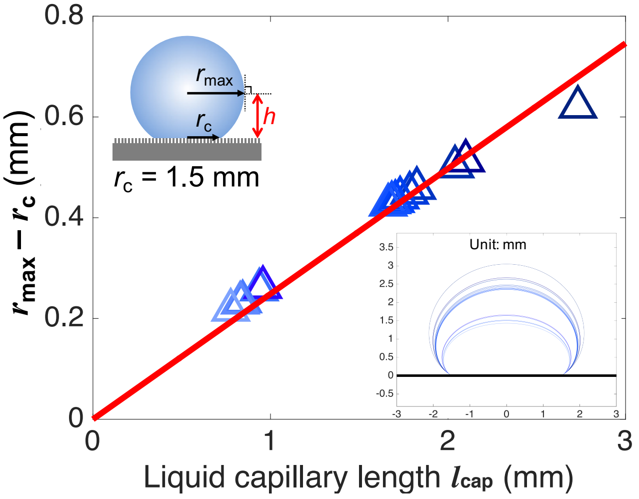

Figure S3. Difference between droplet equator radius and contact radius (r_{max} - r_{c}) vs. capillary length l_{cap} when the contact radius and apparent contact angle are fixed at r_{c} = 1.5 mm and θ^* = 150°. The good linear relation indicates that the meniscus between the solid surface and the droplet equator scales with l_{cap} in the lateral direction. Since the equator height h is also proportional to l_{cap}, the meniscus close to the contact point scales equally in both lateral and vertical directions, leading to the correct lens magnification corresponding to capillary length.

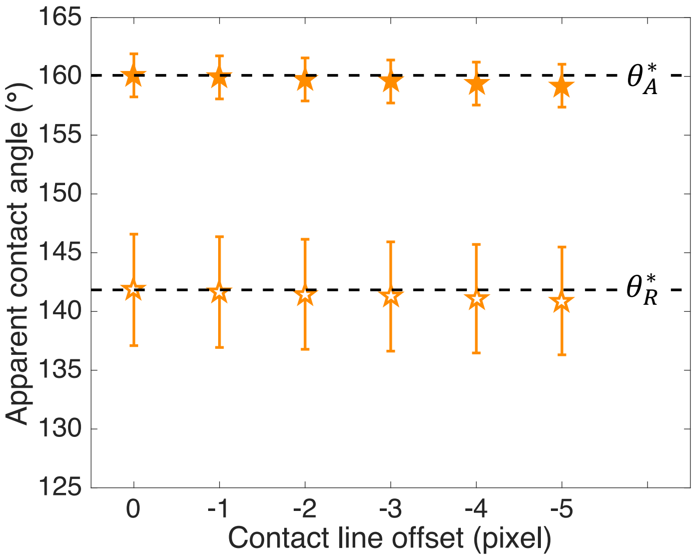

Figure S4. Effect of different contact point selections on the measured apparent contact angles. Images of FC-72 droplets captured at high magnification are analyzed. Due to the high resolution of the captured images, the measured contact angles varied within 1° even when the contact point was manually shifted by 5 pixels above the actual contact point. Such a small variation (< 1°) by the possible pixel error (usually < 2 pixels) is negligible compared with the errors from a wrong magnification setting (~10°). Therefore, the precise determination of a contact point was not a critical factor for accurate measurements of apparent contact angle in the current study. Instead, the main error in reading the apparent contact angles of a small capillary length liquid in a super-repelled state came from the lens magnification, calling for the proposed rule of magnification adjustment.

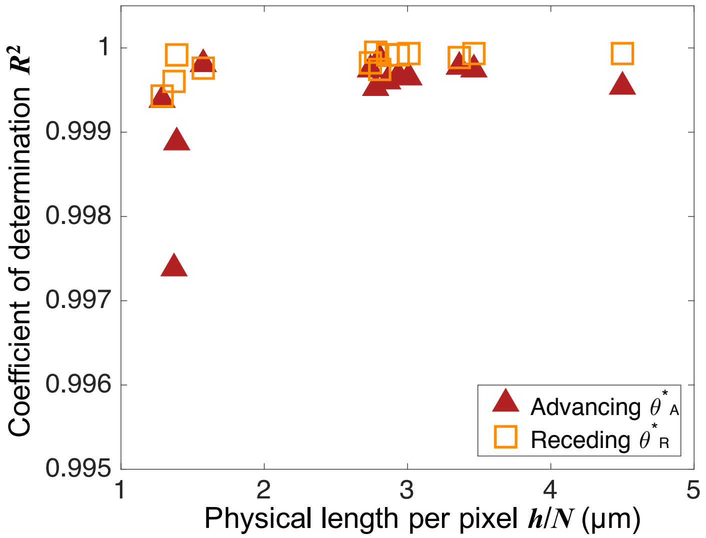

Figure S5. Goodness of polynomial fitting vs. physical length per pixel (h/N) of all physical zoomed apparent contact angle data. The goodness of fitting represented by the coefficient of determination (R^2) is > 0.997 (i.e., extremely good fit) for all pixel sizes. A small physical length per pixel corresponds to a high magnification.

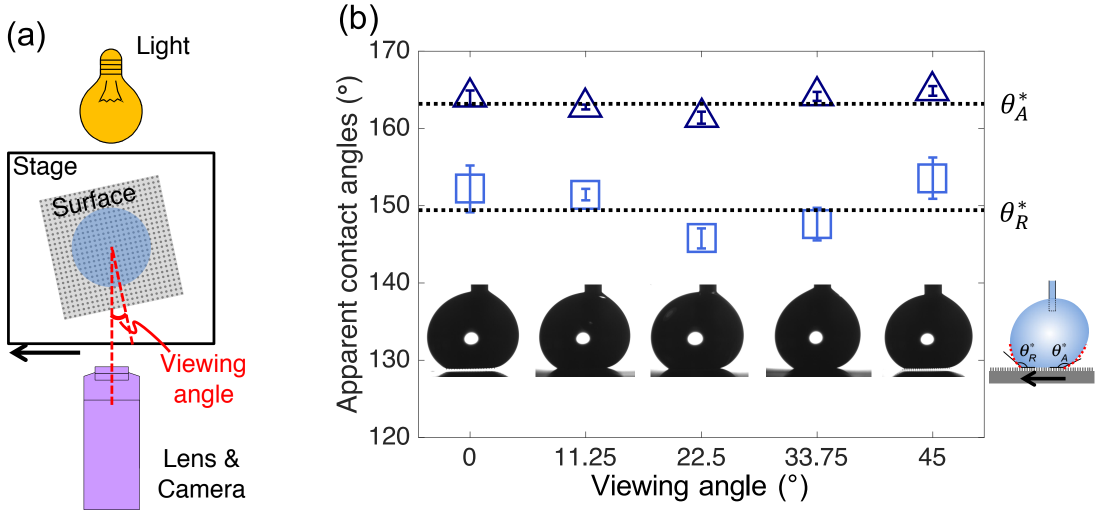

Figure S6. Determination of the best viewing angle to measure the apparent contact angle on a superhydrophobic surface consisting of a square-array of micro-posts. (a) Definition of the viewing angle shown from the top view of the measurement setup schematic. (b) Using water as the test liquid, the apparent contact angles were obtained using 5 different viewing angles within the 45° period representing the square array. Dashed lines indicate the average values of the apparent angles measured from all 5 angles. The apparent contact angles measured at 11.25° (followed closely by 33.75°) are shown to give the values closest to the average of the apparent contact angles observed from all angles. Therefore, we decide to employ the camera viewing angle of 11.25° in all our measurements.
